# Supplementary material for: Bitter taste sensitivity in domestic dogs (Canis familiaris) and its relevance to bitter deterrents of ingestion
Source: PLoS One. 2022 Nov 30;17(11):e0277607. doi: 10.1371/journal.pone.0277607 (PMC9710775; doi:10.1371/journal.pone.0277607)
Supplement: S1 Fig — The tree was generated using the neighbour joining method with Jukes-Cantor protein distance measure and 1,000 bootstrap repetitions. (DOCX) [file pone.0277607.s001.docx]

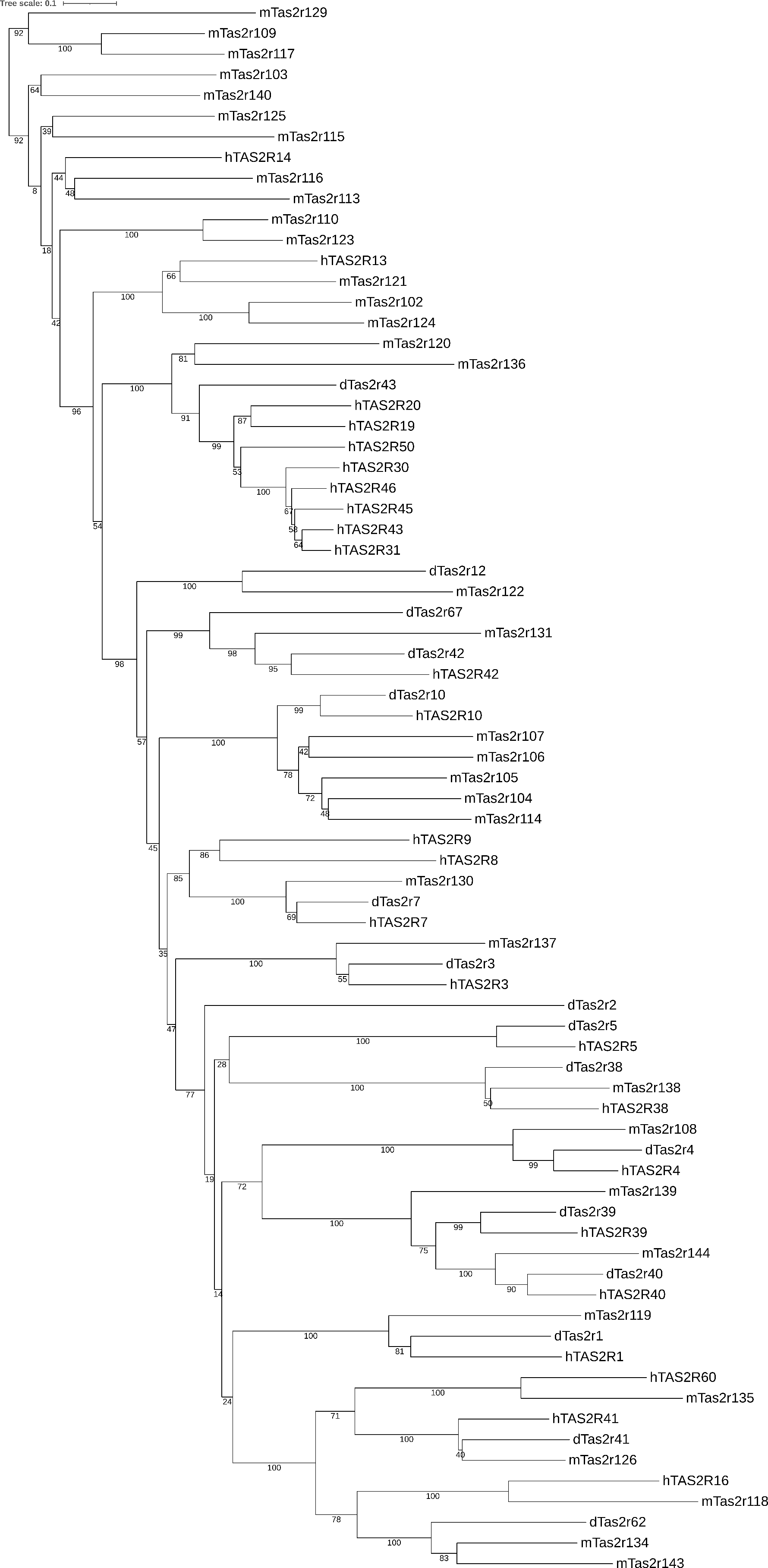


S1 Fig: Phylogenetic tree of all intact human, mouse, and dog Tas2rs. The tree was generated using the neighbour joining method with Jukes-Cantor protein distance measure and 1,000 bootstrap repetitions. Bootstrap values are displayed as node labels.
