# Supplementary material for: Bitter taste sensitivity in domestic dogs (Canis familiaris) and its relevance to bitter deterrents of ingestion
Source: PLoS One. 2022 Nov 30;17(11):e0277607. doi: 10.1371/journal.pone.0277607 (PMC9710775; doi:10.1371/journal.pone.0277607)
Supplement: S2 Fig — Raw data is shown in each case for the highest concentration where a specific response was observed. Concentration-response curves show specific, concentration dependent responses. Where higher concentrations caused high activation in the mock transfects, these have been omitted for clarity. For all experiments n = 2 (error bars = SEM) with the exception of dTas2r2 with ofloxacin where n = 1 (error bars = SD). Compound concentration is plotted on a log scale on the x-axis, the y-axis shows the maximum change in fluorescence divided by the baseline fluorescence before compound injection (ΔF/F0). (DOCX) [file pone.0277607.s002.docx]

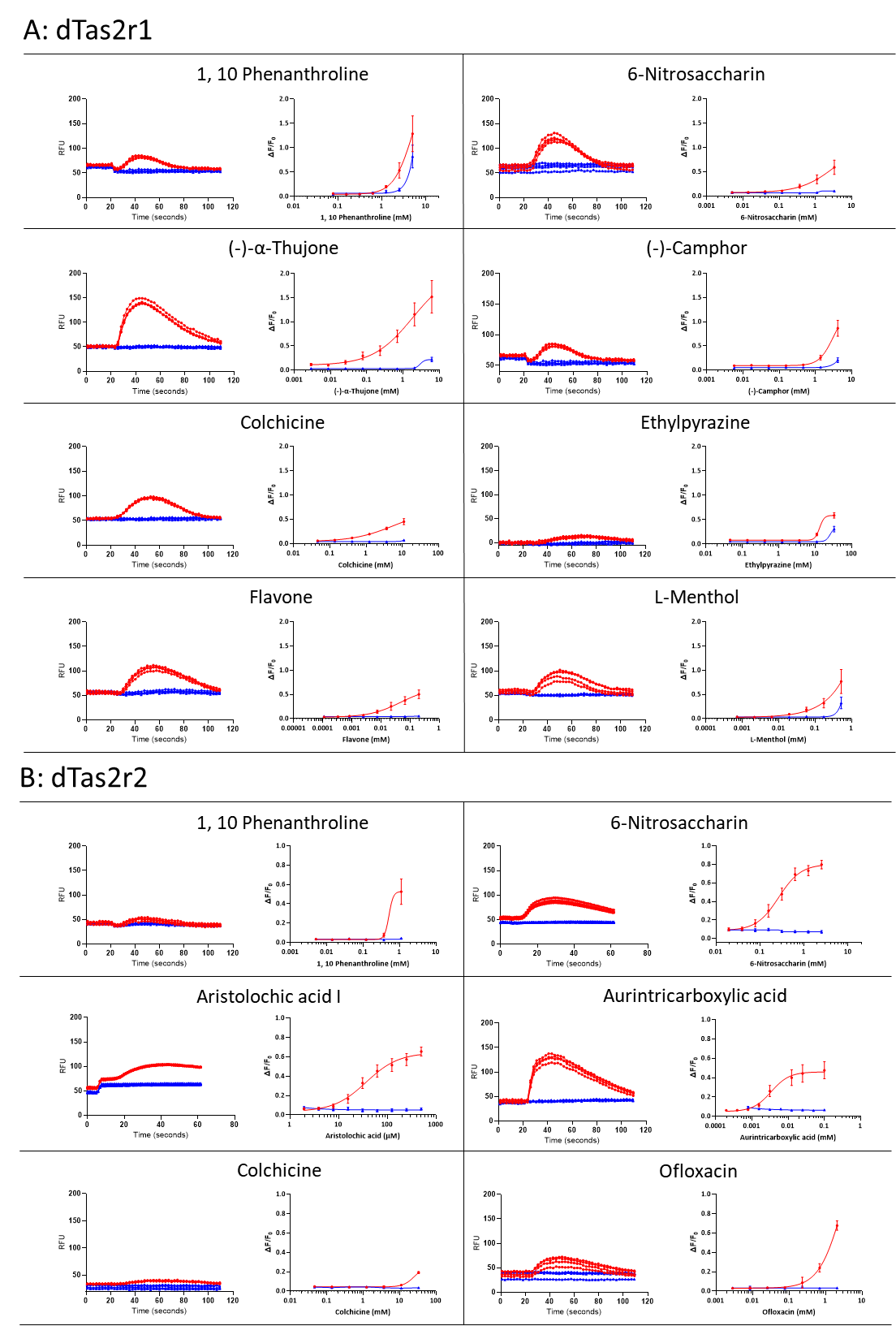


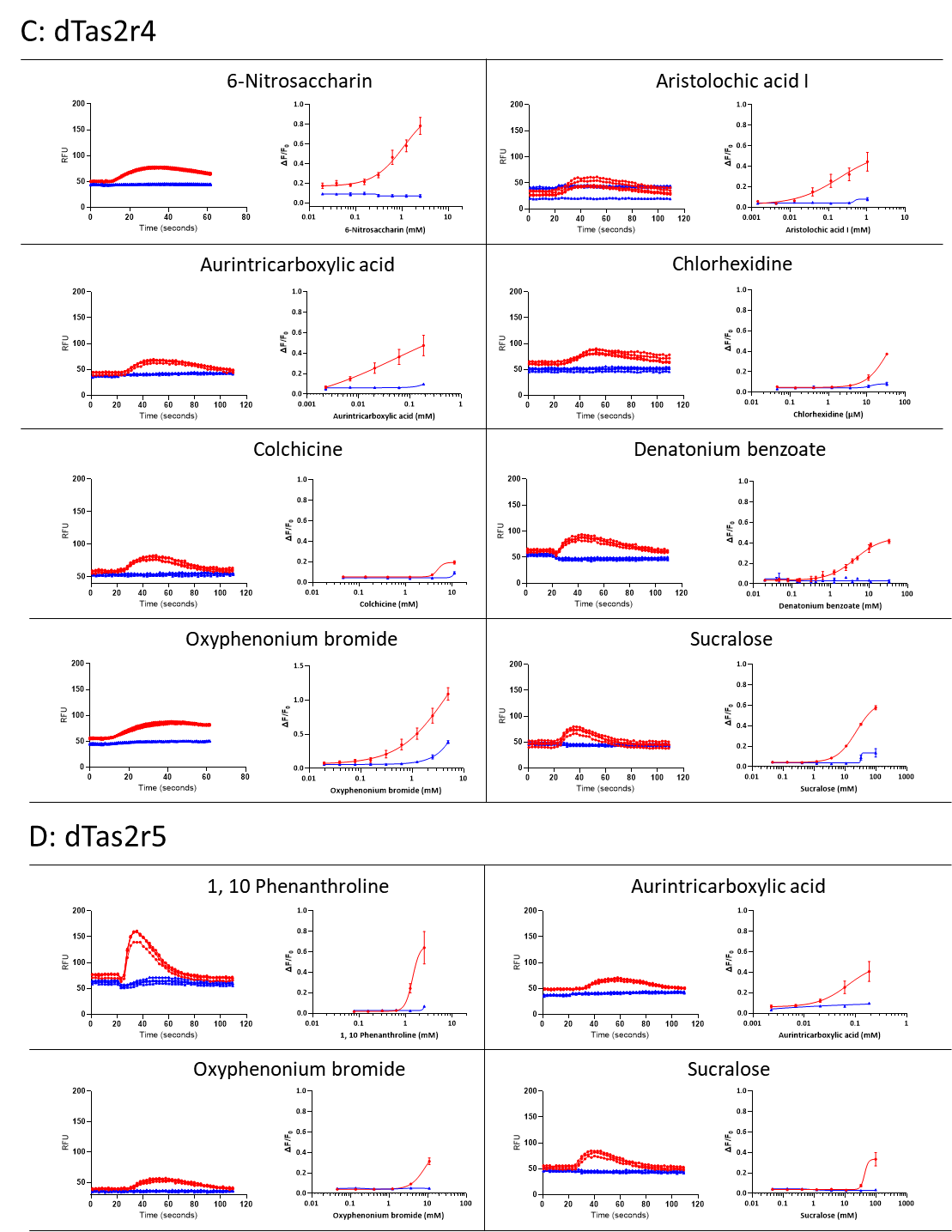


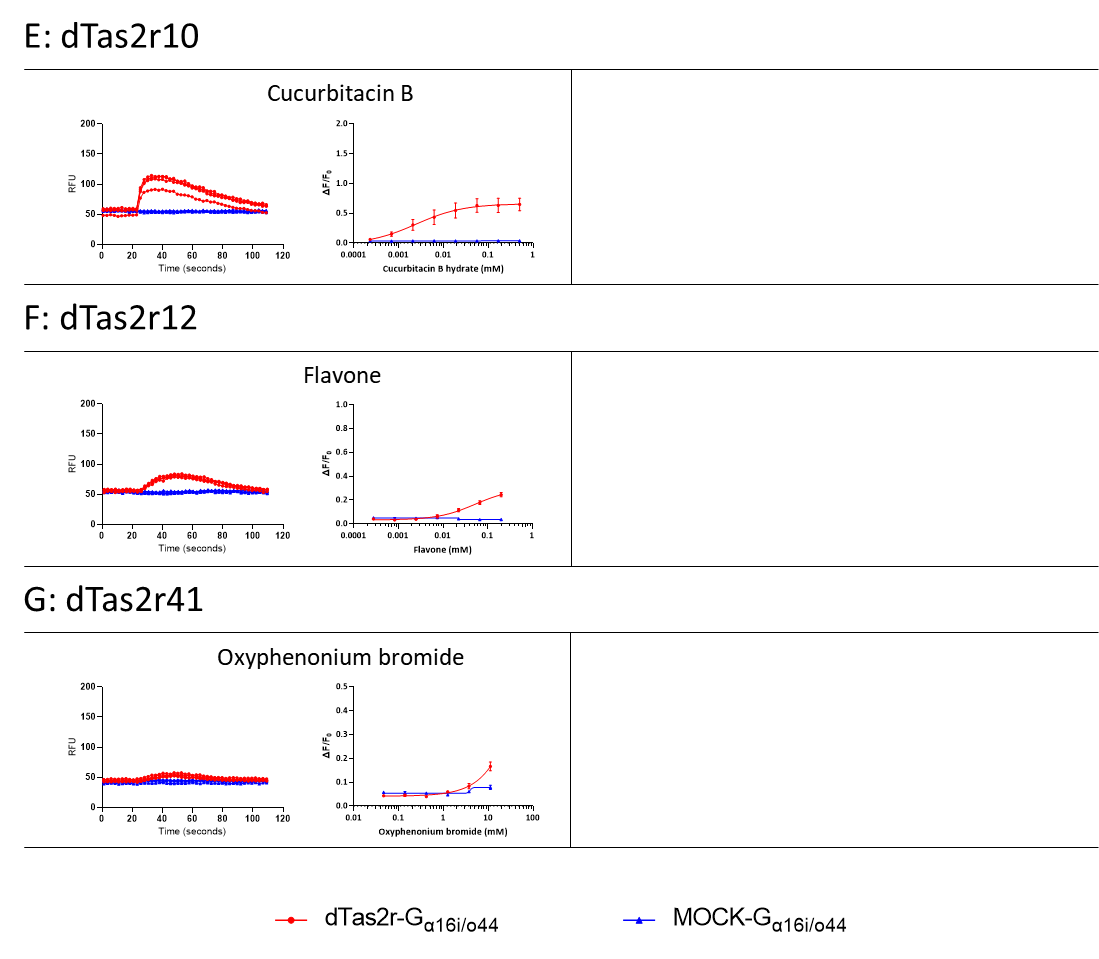


**S2 Fig: All positive receptor-compound combinations.** Raw data is shown in each case for the highest concentration where a specific response was observed. Concentration-response curves show specific, concentration dependent responses. Where higher concentrations caused high activation in the mock transfects, these have been omitted for clarity. For all experiments n=2 (error bars = SEM) with the exception of dTas2r2 with ofloxacin where n = 1 (error bars = SD). Compound concentration is plotted on a log scale on the x-axis, the y-axis shows the maximum change in fluorescence divided by the baseline fluorescence before compound injection (ΔF/F_0_).
