# Supplementary material for: Bitter taste sensitivity in domestic dogs (Canis familiaris) and its relevance to bitter deterrents of ingestion
Source: PLoS One. 2022 Nov 30;17(11):e0277607. doi: 10.1371/journal.pone.0277607 (PMC9710775; doi:10.1371/journal.pone.0277607)
Supplement: S3 Fig — Data are shown as intake difference (g). Data points show each individual exposure event. A) Ten miniature schnauzers were exposed to 10μM DB and plain water on two consecutive days. A significant mean preference for plain water was not apparent (mean = -5.85, 95%CI = -111.89 to 100.19, p = 0.905). B) 76 dogs of three different breeds were exposed to 100μM DB on two consecutive days. A significant mean preference for plain water was seen in the data for all 76 dogs (mean = -244.63, 95%CI = -316.83 to -174.35, p <0.001). Data is also shown separately for the three breeds, cocker spaniels (mean = -355.34, 95%CI = -509.05 and -201.63, p <0.001), Labrador retrievers (mean = -359.41, 95%CI = -561.43 and -157.39, p <0.001), and miniature schnauzers (mean = -112.81, 95%CI = -168.62 and -56.99, p <0.001). A significant mean preference for plain water was seen in all three breeds. Data were assessed using the Tukey post-hoc multiple comparison test. (DOCX) [file pone.0277607.s003.docx]

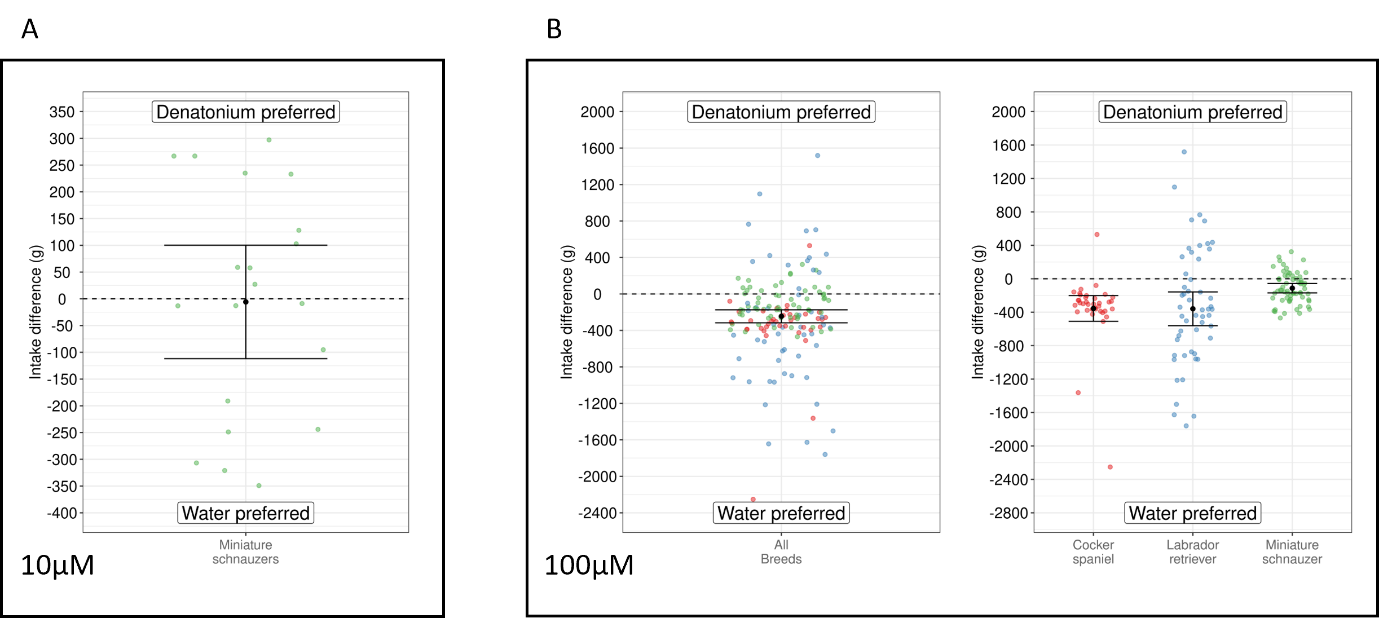


**S3 Fig: Responses of dogs to DB at concentrations of 10μM and 100µM vs plain water.** Data are shown as intake difference (g). Data points show each individual exposure event. A) Ten miniature schnauzers were exposed to 10μM DB and plain water on two consecutive days. A significant mean preference for plain water was not apparent (mean = -5.85, 95%CI = -111.89 to 100.19, *p* = 0.905). B) 76 dogs of three different breeds were exposed to 100μM DB on two consecutive days. A significant mean preference for plain water was seen in the data for all 76 dogs (mean = -244.63, 95%CI = -316.83 to -174.35, *p* <0.001). Data is also shown separately for the three breeds, cocker spaniels (mean = -355.34, 95%CI = -509.05 and -201.63, *p* <0.001), Labrador retrievers (mean = -359.41, 95%CI = -561.43 and -157.39, *p* <0.001), and miniature schnauzers (mean = -112.81, 95%CI = -168.62 and -56.99, *p* <0.001). A significant mean preference for plain water was seen in all three breeds. Data were assessed using the Tukey post-hoc multiple comparison test.
