# Supplementary material for: Bitter taste sensitivity in domestic dogs (Canis familiaris) and its relevance to bitter deterrents of ingestion
Source: PLoS One. 2022 Nov 30;17(11):e0277607. doi: 10.1371/journal.pone.0277607 (PMC9710775; doi:10.1371/journal.pone.0277607)
Supplement: S1 Table — (DOCX) [file pone.0277607.s004.docx]

S1 Table: Bitter compounds used for pre-screening dog Tas2rs.

| **Compound** | **Origin** | **Supplier** | **Product ID** | **CAS Number** |
| --- | --- | --- | --- | --- |
| 1, 10-Phenanthroline | Synthetic | Sigma-Aldrich | 131377-25G | 66-71-7 |
| 4-Benzylpiperidine | Synthetic | Sigma-Aldrich | 142360-25G | 31252-42-3 |
| 6-Nitrosaccharin | Synthetic | CarboSynth | FN15866 | 22952-24-5 |
| Acetaminophen | Synthetic | Sigma-Aldrich | A7085-100G | 103-90-2 |
| 6α-Methylprednisolone | Synthetic | Sigma-Aldrich | M0639-100MG | 83-43-2 |
| Aloin | Natural | Sigma-Aldrich | B6906-25MG | 1415-73-2 |
| (-)-α-Thujone | Natural | Sigma-Aldrich | 89231-5ML | 546-80-5 |
| Aristolochic acid I | Natural | Sigma-Aldrich | A5512-100MG | 313-67-7 |
| Brucine sulphate salt hydrate | Natural | Sigma-Aldrich | B0378-25G | 652154-10-4 |
| (-)-Camphor | Natural | Sigma-Aldrich | 21293-1G | 464-48-2 |
| Chloramphenicol | Natural | Sigma-Aldrich | C0378-5G | 56-75-7 |
| Chlorhexidine | Synthetic | Sigma-Aldrich | 282227-5G | 55-56-1 |
| Chloroquine diphosphate salt | Synthetic | Sigma-Aldrich | C6628-25G | 50-63-5 |
| Colchicine | Natural | Sigma-Aldrich | C9754-1G | 64-86-8 |
| Cucurbitacin B hydrate | Natural | Sigma-Aldrich | C8499-25MG | 6199-67-3 |
| Cycloheximide | Natural | Sigma-Aldrich | 01810-5G | 66-81-9 |
| Denatonium benzoate | Synthetic | Sigma-Aldrich | D5765-10G | 3734-33-6 |
| Dextromethorphan hydrobromide monohydrate | Synthetic | Sigma-Aldrich | D2531-5G | 6700-34-1 |
| 1,1-Dimethylbiguanide hydrochloride | Synthetic | Sigma-Aldrich | D150959-5G | 1115-70-4 |
| 1,1-Diphenyl-4-piperidino-1-butanol hydrochloride (Diphenidol) | Synthetic | Tokyo Chemical Industry (TCI) | D2062 | 3254-89-5 |
| Doxepin hydrochloride | Synthetic | Sigma-Aldrich | D4526-1G | 1229-29-4 |
| Ethylpyrazine | Natural | Sigma-Aldrich | 250384-5G | 13925-00-3 |
| Flavone | Natural | Sigma-Aldrich | F2003-1G | 525-82-6 |
| N-(3-Oxooctanoyl)-L-homoserine lactone | Natural | Sigma-Aldrich | O1764-100MG | 147795-39-9 |
| Aurintricarboxylic acid | Synthetic | Sigma-Aldrich | A1895-25G | 4431-00-9 |
| L-Menthol | Natural | Sigma-Aldrich | W266523-100G | 2216-51-5 |
| Ofloxacin | Synthetic | Sigma-Aldrich | O8757-10G | 82419-36-1 |
| Oleuropein | Natural | Sigma-Aldrich | 12247-50MG | 32619-42-4 |
| Omeprazole | Synthetic | Sigma-Aldrich | O104-100MG | 73590-58-6 |
| Oxybutynin chloride | Synthetic | Sigma-Aldrich | O2881-5G | 1508-65-2 |
| Oxyphenonium bromide | Synthetic | Sigma-Aldrich | O5501-5G | 50-10-2 |
| Papaverine hydrochloride | Natural | Sigma-Aldrich | P3510-5G | 61-25-6 |
| Parthenolide | Natural | Sigma-Aldrich | P0667-5MG | 20554-84-1 |
| Picrotoxin | Natural | Sigma-Aldrich | P1675-5G | 124-87-8 |
| Pirenzepine dihydrochloride | Synthetic | Sigma-Aldrich | P7412-1G | 29868-97-1 |
| Prednisone | Synthetic | Sigma-Aldrich | P6254-10G | 53-03-2 |
| 6-Propyl-2-thiouracil (PROP) | Synthetic | Sigma-Aldrich | P3755-10G | 51-52-5 |
| N-Phenylthiourea (PTC) | Synthetic | Sigma-Aldrich | P7629-10G | 103-85-5 |
| Quinacrine dihydrochloride | Synthetic | Sigma-Aldrich | Q3251-25G | 69-05-6 |
| Quinine hydrochloride dihydrate | Natural | Sigma-Aldrich | Q1125-5G | 6119-47-7 |
| Resveratrol | Natural | Sigma-Aldrich | R5010-100MG | 501-36-0 |
| Sucralose | Synthetic | Sigma-Aldrich | 69293-100G | 56038-13-2 |
| D-(-)-Salicin | Natural | Sigma-Aldrich | S0625-25G | 138-52-3 |
| Sinigrin hydrate | Natural | Sigma-Aldrich | 85440-1G | 3952-98-5 |
| Strychnine hydrochloride | Natural | Sigma-Aldrich | S8753-25G | 1421-86-9 |
| Thiamine hydrochloride | Natural | Sigma-Aldrich | T4625-5G | 67-03-8 |
| Trimethoprim | Synthetic | Sigma-Aldrich | 92131-5G | 738-70-5 |
| Yohimbine | Natural | Sigma-Aldrich | Y3125-10G | 65-19-0 |
