# Supplementary material for: Bitter taste sensitivity in domestic dogs (Canis familiaris) and its relevance to bitter deterrents of ingestion
Source: PLoS One. 2022 Nov 30;17(11):e0277607. doi: 10.1371/journal.pone.0277607 (PMC9710775; doi:10.1371/journal.pone.0277607)
Supplement: S2 Table — Substituted amino acids are highlighted in yellow with bold text. (DOCX) [file pone.0277607.s005.docx]

**S2 Table**: **Dog and human Tas2r10 chimeric receptor sequences.** Substituted amino acids are highlighted in yellow with bold text.

| Regions | Sequence |
| --- | --- |
| Chimera1  dog Tas2r10 with human EC2 | MLSILEGLLIFIAVSESILGVLGNGFIGLVNCIDCVKNKKFSMVGFILTGLATSRICLILIIITDGFIKIFSPDMYSSGNLIDYISYLWVIINQSSIWFATSLSIFYFLKIANFSHHIFLWLKGRINSVLPLLMGSLFISWLFTFPQIVKI**L**ND**YKT**K**‑ND**T**V**W**D**LNM**Y**KSE**Y**F**I**KQILLNLGVILLFTLCLITCFLLIVSLWRHNRHMQLNVTGLRDPSTEAHVKAMKILVSFIILFILYFIGIAIEISCFILPENKLLFIFGMMTTAIYPWGHSFILILGNSKLKQASLKTLQQLKCEARRLLTAAQIHVGGNGCSRRII |
| Chimera2 dog Tas2r10 with human ECL1 and TM3 | MLSILEGLLIFIAVSESILGVLGNGFIGLVNCIDCVKNKKFSMVGFILTGLATSRICLILIIITDGFIKIFSP**NI**Y**A**SGNLI**E**YISY**F**WVI**G**NQSS**M**WFATSLSIFYFLKIANFSHHIFLWLKGRINSVLPLLMGSLFISWLFTFPQIVKIINDNRMKSRNTTWQLNMQKSEFFTKQILLNLGVILLFTLCLITCFLLIVSLWRHNRHMQLNVTGLRDPSTEAHVKAMKILVSFIILFILYFIGIAIEISCFILPENKLLFIFGMMTTAIYPWGHSFILILGNSKLKQASLKTLQQLKCEARRLLTAAQIHVGGNGCSRRII |
| Chimera3  dog Tas2r10 with human TM5 and Q206 of IC3 | MLSILEGLLIFIAVSESILGVLGNGFIGLVNCIDCVKNKKFSMVGFILTGLATSRICLILIIITDGFIKIFSPDMYSSGNLIDYISYLWVIINQSSIWFATSLSIFYFLKIANFSHHIFLWLKGRINSVLPLLMGSLFISWLFTFPQIVKIINDNRMKSRNTTWQLNMQKSEFFTKQILLNLGVI**FF**FTL**S**LITC**IF**LI**I**SLWRHNR**Q**MQLNVTGLRDPSTEAHVKAMKILVSFIILFILYFIGIAIEISCFILPENKLLFIFGMMTTAIYPWGHSFILILGNSKLKQASLKTLQQLKCEARRLLTAAQIHVGGNGCSRRII |
| Chimera4  dog Tas2r10 with human TM6 | MLSILEGLLIFIAVSESILGVLGNGFIGLVNCIDCVKNKKFSMVGFILTGLATSRICLILIIITDGFIKIFSPDMYSSGNLIDYISYLWVIINQSSIWFATSLSIFYFLKIANFSHHIFLWLKGRINSVLPLLMGSLFISWLFTFPQIVKIINDNRMKSRNTTWQLNMQKSEFFTKQILLNLGVILLFTLCLITCFLLIVSLWRHNRHMQLNVTGLRDPSTEAHVKAMK**V**L**I**SFIILFILYFIG**M**AIEISCFILPENKLLFIFGMMTTAIYPWGHSFILILGNSKLKQASLKTLQQLKCEARRLLTAAQIHVGGNGCSRRII |
| Chimera5  dog Tas2r10 with human EC3 and TM7 | MLSILEGLLIFIAVSESILGVLGNGFIGLVNCIDCVKNKKFSMVGFILTGLATSRICLILIIITDGFIKIFSPDMYSSGNLIDYISYLWVIINQSSIWFATSLSIFYFLKIANFSHHIFLWLKGRINSVLPLLMGSLFISWLFTFPQIVKIINDNRMKSRNTTWQLNMQKSEFFTKQILLNLGVILLFTLCLITCFLLIVSLWRHNRHMQLNVTGLRDPSTEAHVKAMKILVSFIILFILYFIGIAIEISCF**TVR**ENKLL**LM**FGM**T**TTAIYPWGHSFILILGNSKLKQASLKTLQQLKCEARRLLTAAQIHVGGNGCSRRII |
| Chimera6  human TAS2R10 with dog EC2 | MLRVVEGIFIFVVVSESVFGVLGNGFIGLVNCIDCAKNKLSTIGFILTGLAISRIFLIWIIITDGFIQIFSPNIYASGNLIEYISYFWVIGNQSSMWFATSLSIFYFLKIANFSNYIFLWLKSRTNMVLPFMIVFLLISSLLNFAYIAKI**I**ND**NRM**K**SRN**T**T**W**Q**LNM**Q**KSE**F**F**T**KQILLNLGVIFFFTLSLITCIFLIISLWRHNRQMQSNVTGLRDSNTEAHVKAMKVLISFIILFILYFIGMAIEISCFTVRENKLLLMFGMTTTAIYPWGHSFILILGNSKLKQASLRVLQQLKCCEKRKNLRVT |
