# Supplementary material for: Bitter taste sensitivity in domestic dogs (Canis familiaris) and its relevance to bitter deterrents of ingestion
Source: PLoS One. 2022 Nov 30;17(11):e0277607. doi: 10.1371/journal.pone.0277607 (PMC9710775; doi:10.1371/journal.pone.0277607)
Supplement: S3 Table — Alternative or previous gene numbering shown in brackets (suffix p = pseudogene). (DOCX) [file pone.0277607.s006.docx]

S3 Table: Previously reported *Tas2rs* in the dog and those identified in this study. Alternative or previous gene numbering shown in brackets (suffix p = pseudogene).

| Go and Investigators, 2006 (49). | Dong D, Jones G, Zhang S, 2009 (50). | Hu LL, Shi P, 2013 (51). | Shang S, Wu X, Chen J, Zhang H, Zhong H, Wei Q, *et al*, 2017(52) | **This study** |
| --- | --- | --- | --- | --- |
| *Tas2r1* | *Tas2r1* | *Tas2r1* | *Tas2r1* | *Tas2r1* |
| *Tas2r2* | *Tas2r2* | *Tas2r2* | *Tas2r2* | *Tas2r2* |
| *Tas2r3* | *Tas2r3* | *Tas2r3* | *Tas2r3* | *Tas2r3* |
| *Tas2r4* |  | *Tas2r4 (16)* | *Tas2r4p* | *Tas2r4* |
| *Tas2r5* | *Tas2r5* | *Tas2r5* | *Tas2r5* | *Tas2r5* |
| *Tas2r7* | *Tas2r7* | *Tas2r7* | *Tas2r7* | *Tas2r7* |
| *-* | *-* | *-* | *Tas2r7 like 1p* | *-* |
| *-* | *-* | *-* | *Tas2r7 like 2p* | *-* |
| *Tas2r8p* | *-* | *-* | *Tas2r8p* | *Tas2r8p* |
| *Tas2r9p* | *Tas2r9* | *Tas2r9* | *Tas2r9p* | *Tas2r9p* |
| *Tas2r10* | *Tas2r10* | *Tas2r10* | *Tas2r10* | *Tas2r10* |
| *Tas2r12* | *Tas2r12* | *Tas2r12 (4)* | *Tas2r12* | *Tas2r12* |
| *-* | *-* | *-* | *Tas2r19p* | *-* |
| *Tas2r31p (44)* | *-* | *-* | *Tas2r31p (44)* | *Tas2r31p (44)* |
| *-* | *-* | *-* | *Tas2r34* | *-* |
| *Tas2r38* |  | *Tas2r38 (8)* | *Tas2r38* | *Tas2r38* |
| *Tas2r39* | *Tas2r39* | *Tas2r39 (11)* | *Tas2r39* | *Tas2r39* |
| *Tas2r40* | *Tas2r40* | *Tas2r40 (12)* | *Tas2r40* | *Tas2r40* |
| *Tas2r41* | *Tas2r41* | *Tas2r41 (13)* | *Tas2r41* | *Tas2r41* |
| *Tas2r42 (55)* | *Tas2r42 (55)* | *Tas2r42 (15)* | *Tas2r42* | *Tas2r42* |
| *Tas2r43* | *Tas2r43* | *Tas2r43 (14)* | *Tas2r43* | *Tas2r43* |
| *Tas2r62p* | *Tas2r62* | *-* | *Tas2r62p* | *Tas2r62* |
| *Tas2r67* | *Tas2r67* | *Tas2r67 (6)* | *Tas2r67* | *Tas2r67* |
| Totals (pseudogenes) | | | |  |
| 15 (4) | 15 (0) | 16 (0) | 15 (8) | 16 (3) |
