# Supplementary material for: Bitter taste sensitivity in domestic dogs (Canis familiaris) and its relevance to bitter deterrents of ingestion
Source: PLoS One. 2022 Nov 30;17(11):e0277607. doi: 10.1371/journal.pone.0277607 (PMC9710775; doi:10.1371/journal.pone.0277607)
Supplement: S4 Table — (DOCX) [file pone.0277607.s007.docx]

**S4 Table: Dog Tas2r sequences identified and used in this study.**

| dTas2r1 | MLEFYLIIHFLFTVMQFLIGVLANGIIVVVNGTELIKQRKMIPLALLLCCLAISRICLQLIIFFMNLGTLFLIEVPLLADNFVIFVFVNELGLWFATWLGVYYCAKIAPITHSFFFWLKIRISKWMPWLILGSMMYASVPSVFCSKQIWVYSQNVLSSLFSPNATQIKETSALQIAFLIRLLLPLLIFLGSTLLLIFSLGRHTWQMRNTATGPRDPSTGVHVSTILSVLSFLVLCLSHYMAAALLSFQIFQLRSLVFLICLWVFGSYPSGHSMILILGNPKLKQNAKKLLLHGKCCQ |
| --- | --- |
| dTas2r2 | MISFLSALPHVIVMSAEFITGITVNGFLIIMNCKELIKSRKPTPVQLLFICIGMSRFGLLMVLMIQSFFSVLFPLFYKVNIFGTAMLFFWMFFSSVSFWFATCLSVFYCLKIAGFTQSCFLWLKFRISKLMPWLLLGSLLASMSIAALCIEADYPKKVDDDALKNATLKRTEPKIRQISEMLLVNLALLFPLAIFVMCTFMLFISLYKHTHRMQNGSHGVRNASTKAHINALKTVITFFCFFISYFAAFMANMTFSIPYGSHCFFVVKDIMAAFPSGHSIIILLSNSKYQQPFRRLLCFKKNQ |
| dTas2r3 | MSGLGKSVFLVLSVTQFILGMLGNGFIVLVNGSSWFKNKTVSLSDVIITNLALSRIVLLWILLVDGVLMVFFSKVHDEGTVMEIIDIFWTFTNHLSIWLATCLSVLYCLKIASFSHPTFLWLKWRVSRVVVQMILGALLLSCASAMSLVHEFKIYSILSGIAGTGNVTEHFRKKRNDYKVAHVLGTLWNLPPLIVSLASYFLLIFSLGRHTQQMKHSGTSSRDLSTEAHQRAIKIIVSFLFLFLLYFLAFLITSSSYFIPETEMVKRVGVVVTMFYPASHSFVIILGNNKLKQMFTEMLCCEPGYLKPGFKRPFAP |
| dTas2r4 | MLQIFFLSAIIFSAILNFVGLIVNLFIAVVSYRTWLKSHRISSSNWILFSLGITRFLMLGLFLLNIIYFFISPKMERSVHLSHFFLSCWMFLDSNSLWFVTLLNALYCVKITDFQLGVFLLLKRNLSPKIPRLLLACVLISAFTTLLYVVLKQTSSLPEFVTQRNGTGCGIHGSVLSLVTSLVLRSVLQFIINVTSASLLIHSLRRHIQKMQKNTTIFWNPQTEAHVGAMKLMICFLILYIPYSVATLLHYFPYGGMDLRTRSICLVISSFYPPGHSILIILTHPKLKTKAKKILCFNK |
| dTas2r5 | MLTAALPLLMVVAVVEFLIGLVGNGVLMVWSFGEWVRKFNGSSYNLIVLGLAVCRFLLQCLIMMDLSLFPFFQSSRWLHYLSIFWILVSQASLWFATFLSVFYCRKIMTLEHPVCLWLKQRAYCLSLWCLLVYLMISLLLVAHIGLKPYNPSQGNSSILYPLKSWHYLYIVKLNAGSGLPLMVFLVSSGMLIVSLYRHHKKMEVHTAGRRDAQAKAHITVLKSLGCFLILHVIYILASPFSITSKSSADLLVVFISETVMAAYPSLHSVILILGNPRMKQTCQRILWKTVCAWKS |
| dTas2r7 | MPDKVESILMLVAAGEFSMGILGNTFIGLVNCIGWIKKRKIASIDLILTSLAISRICLLCIILLDCFILVLYPDVYATGKQMRIIDFFWTLTNHLSVWFATCLSIFYFLKIANFFHPLFLWMKWRIDSAIPRILLGCLALSVFISLVVTENLNDDFRCCVRTKKKTNLTVRCRVKKAKYSSIKICLNLLTLFPFSVSLISFLLLILSLWRHTRQMKFNATGCRDFSIEAHMGAMKAVISFLLLFIAYYLAFLVATSSYFMPETELAVIIGELIALIYPSSHSFILILGSNKLRQASLRVLWKVKYVLKRRNF |
| dTas2r10 | MLSILEGLLIFIAVSESILGVLGNGFIGLVNCIDCVKNKKFSMVGFILTGLATSRICLILIIITDGFIKIFSPDMYSSGNLIDYISYLWVIINQSSIWFATSLSIFYFLKIANFSHHIFLWLKGRINSVLPLLMGSLFISWLFTFPQIVKIINDNRMKSRNTTWQLNMQKSEFFTKQILLNLGVILLFTLCLITCFLLIVSLWRHNRHMQLNVTGLRDPSTEAHVKAMKILVSFIILFILYFIGIAIEISCFILPENKLLFIFGMMTTAIYPWGHSFILILGNSKLKQASLKTLQQLKCEARRLLTAAQIHVGGNGCSRRII |
| dTas2r12 | MAGTMKNVFMMIFAGEFIIGILGNGFIILVNCIDWIRSWKFFLIDFILTCLAISRIFLLCIIMLGIGLDIICKEIWYNDNQLITFEVLWTGCNYFCTICTVCLSVFYFLKIANSSNPIFFWLKRRIHRLLLIIVLGAVFYFCLSLLLKDIVFKNMIKTKVNTESNVTLNFTARKYDLLTSNIFLNMLFVIPFAVSLASFVLLIHSLWNHTRRMKGIDSGDLITEAHVRAMKFMISFLLFFFIYYLSNIIIYFAYVVLDSLVAKIFANILVFSYPSGHPFLLILWNCKLKQASLYVLRKLKWCMNLRKPAYIKHT |
| dTas2r38 | MLALTPVITVSYEVKSAFMFLSVLELAVGILTNAFIFLVNFWDVVRRQPLSNCDLILLSLSLTRLFLHGLLFLDAIQLTYFQRMKDPLSLSYQTIIMLWMITNQAGLWLTTCLSLFYCSKIVRFSHTLLLCLANWVSRKAPQMLLGAMLFSSACTLLCLGDFFSRSGFAFTTVLLMNNTEFNSQIVKLNFYYSSIFCTLGSIPPFMFFLVSSGVLIISLGRHMRTMKANTKDSGDPSLEAHIKALISLISFLCLYVVSFCVALISVPLTMVWHNKIGVMICVGILAACPSIHAAILISGNAKLRRAVETILLWVQSSLKVRAGHRADLRTPDLC |
| dTas2r39 | MMETCNPPENELSPFGILSILTITGTECIVGIIANGFIMAINAAEWIKNKTVSTSGRVLFFLSASRIALQSFTMLEITFSSTSPRFYNEDVMYDTFKVSFMFLNHCSLWFAAWLSFFYFVKIADFSHPLFLKLKWRISRLMPWLLWLSVLISLGYSMLLSNDIYTVYCNNSSIPSSNSTKKKYFTKTNVVNLVLLYNLGIFIPLIMFILSATLLIISLKRHTLHMESNATGCRDPSMEAHIGAIRATSYFLILYIFNSVALFLYMSNIFDINSSWNILCKFIMAAYPAGHSILLIQDNPGLRRAWKRLQPQVHFYLKEQTP |
| dTas2r40 | MATVSTDATDRDMSRFKIVLTLVVPGIECLTGIVGNGFITIIHGAKWARGKRLPVTDCILLMLSFSRLLLQIWMMLENIYSLLFRVTYNQSTVFIVFKVTVIFLNYFNLWLAAWLNIFYCLRITNLAHHVFFMMKRKITELMPRLLGLSLFISLCFSFPFSTDIFHVYVNSSIPIRSSNTTEKKYFSETNVVNLVLLYNLGIFIPLIMFILSATLLIISLKRHTLHMESNATGCRDPSMEAHFGAIRATSYFLILYIFNAVALFLSMSNIFDINSSWNILCKIVMAAYPASHSVLLILGNPGLRRAWKRFQHHVPLHL |
| dTas2r41 | MQPAVSAFFMLLFVLLCVLGILANGFIVLVLSRERMRRGRLLPSDVILLSLGASRFCLQCIGMMNNFYYYLHLEEYSTGPARQFFGLHWDFLNSATFWFGSWLSVLFCMKIASFTHPTFLWLRWRLPGSVPWLLGASLLISFLVTLLFFWGNHAVYQGFLIRKYPGNMTFQQWSRRLEIHYFLPLKFITLSVPCSVFLVSIALLINSLRRHRGRMRRSGHGLQDPSSQAHTRALKSLVSFLILYALSFASLVIDAAGFFCSQSDWYWPWQILIYLCTSVHPYILILSNLRLRGGCRQLLLLVRGSQLA |
| dTas2r42 | MLAGLDIIFLTLSTAEFIIGMLGNAFIGLVNCSEWVKNRKISLADFILICLAISRIAQLLVSWFESFMMGLSPLFFSTYKLAKSITLLWRITHHLATWFSTCLSIFYLLKIAQFSHSLFLWLRWRMNRVVLAILVFSLFFLLFDFLMLETFNDLFSNVDAMDESNLTLYIYESKTFYVKTLILLSFSYIIPIILSLTSLLLLFLSLVKHIRNLQLNSMGSRDSSTQAHKKAIKMVMSFLFLFTVHFFSIQLSNWMFFLFWNKKITKFIMLAVYVFPSSHSLILILGNSKLRQTALKVLWHLKSSLKREKPNSSLPIDFPESFQ |
| dTas2r43 | MLPLLQSIFSILVMTEFVLGNFANGFIVLVNYIAWVKRQKISSADQILTGLAVSRIGLLWVILINWYATLLNPALYSLEVRLLVHIAWTANNHFSIWLATSLSVFYLFKIANFSNLIFLRLKWRVKSVVFVMLLGSLFFLVFHVAVVSIYEQMQMKEYEGNITRQTKLRDIAQLMNMTVFTLMNFVPFAISLTSFLLLIFSLWKHLKKMRSGGKRYQDSSTKVHIKAMQTVISFLLLLVCYFLTLIAIVWSSNRLQNKLIFLLCKAIGILYPSSHSFILIWGNKKLREDFLSFLWQLKGWLKKGYKRSIMCLLGENKLMESVIFFSSTSFSNEYVIEQFPKIYLKKSFL |
| dTas2r62 | MSSSPTLIFMVIFFLESLAAMLQNGFMVTVLGREWVRRRTLPAGDMIVASLAASWFCLHGVAILNNLLIFFGFHFVRDYYNTLWHFVNTLTLWLTAWLAVFYCVKVAVFSHPVFFWLKWRISRLVPRLLLGSLVLVGLTVISSAIVTGILKQMIASKSSQGNSTWAERVQAFYRSFHLFDVMLMWSVPFLLFLVSMLLLVFSLCRHLGLMRNYRQDPCDPSTRVHTMALKSLVFFLVFYTPYFLSLVVVAIEITNFQSHWYWAWEVVTYASICLHSSMLVLSSPKLRKVLMTRLWKALDKG |
| dTas2r67 | MPSRIENAFLVAAAGELITGMLGNGFIVLVNCIDLVKNLKLSTADCILTSLALSRIILLCIILLDSLLMVFWQHLYAIDKLAKFISVFWTLSNHLTTWIVTCLNVFYFFKIANFSHPCFTWLRWRISRVLLVLPLGSLFLLFFNFELLDTFTNFWVNLYQRHERNSIWSLDVSKTLYLNSLIVFSFIYLIPFLLSLASLLLLFLSLMRHIRNVQRNSSSRDFRTEAHKRAMKMVMSSLFLSMVNFTSILLTGWFSLLLQNHQANLAVLLLSTLVPSGHSFILILGNNKLRQAALGLLWHLNCHLKMVKPFAS |
