# Supplementary material for: Bitter taste sensitivity in domestic dogs (Canis familiaris) and its relevance to bitter deterrents of ingestion
Source: PLoS One. 2022 Nov 30;17(11):e0277607. doi: 10.1371/journal.pone.0277607 (PMC9710775; doi:10.1371/journal.pone.0277607)
Supplement: S5 Table — (DOCX) [file pone.0277607.s008.docx]

S5 Table: Compounds selected for full concentration-response testing with dog Tas2rs based on pre-screen testing.

| **Receptor** | **Compounds selected for full concentration-response testing** |
| --- | --- |
| dTas2r1 | 1, 10 Phenanthroline, 6-Nitrosaccharin, 6α-Methylprednisolone, Aloin, (-)-α-Thujone, (-)-Camphor, Chloramphenicol, Chloroquine, Colchicine, Cycloheximide, Denatonium benzoate, Dextromethorphan hydrobromide, Dimethylbiguanide, Diphenidol, Doxepin, Ethylpyrazine, Flavone, Aurintricarboxylic acid, L-Menthol, Ofloxacin, Oxyphenonium bromide, Parthenolide, Picrotoxinin, Resveratrol, Sucralose, Sinigrin, Thiamine, Yohimbine |
| dTas2r2 | 1, 10 Phenanthroline, Colchicine, Denatonium benzoate, Diphenidol, Aurintricarboxylic acid, Ofloxacin |
| dTas2r3 | 6-Nitrosaccharin, 6α-Methylprednisolone, (-)-α-Thujone, Aristolochic acid I, (-)-Camphor, Chloramphenicol, Chlorhexidine, Chloroquine, Colchicine, Cucurbitacin B, Cycloheximide, Diphenidol, Ethylpyrazine, Flavone, N-(3-Oxooctanoyl)-L-homoserine lactone, 6-Propyl-2-thiouracil |
| dTas2r4 | Aloin, (-)-α-Thujone, Aristolochic acid I, (-)-Camphor, Chloramphenicol, Chlorhexidine, Colchicine, Cucurbitacin B, Cycloheximide, Denatonium benzoate, Diphenidol, Flavone, N-(3-Oxooctanoyl)-L-homoserine lactone, Aurintricarboxylic acid, Parthenolide, Picrotoxinin, Prednisone, Quinine, Resveratrol, Sucralose, Yohimbine |
| dTas2r5 | 1, 10 Phenanthroline, 6α-Methylprednisolone, Aloin, (-)-α-Thujone, (-)-Camphor, Chloramphenicol, Chlorhexidine, Chloroquine, Colchicine, Cucurbitacin B, Denatonium benzoate, Diphenidol, Flavone, N-(3-Oxooctanoyl)-L-homoserine lactone, Oxyphenonium bromide, Sucralose |
| dTas2r7 | 6α-Methylprednisolone, Aloin, (-)-α-Thujone, (-)-Camphor, Chloramphenicol, Chlorhexidine, Chloroquine, Colchicine, Cucurbitacin B, Cycloheximide, Denatonium benzoate, Diphenidol, Ethylpyrazine, Flavone, N-(3-Oxooctanoyl)-L-homoserine lactone, Aurintricarboxylic acid, Oxyphenonium bromide, Papaverine, Resveratrol, Sucralose |
| dTas2r10 | (-)-α-Thujone, Aristolochic acid I, (-)-Camphor, Chloramphenicol, Chloroquine, Cucurbitacin B, Cycloheximide, Denatonium benzoate, Dextromethorphan hydrobromide, Diphenidol, Ethylpyrazine, Oxybutynin chloride, Oxyphenonium bromide, Papaverine, Parthenolide, Picrotoxinin, Pirenzepine, Quinine, Sucralose, Strychnine, Yohimbine |
| dTas2r12 | Chloramphenicol, Cucurbitacin B, Diphenidol, Flavone |
| dTas2r38 | Aloin, (-)-α-Thujone, (-)-Camphor, Chloramphenicol, Chlorhexidine, Chloroquine, Colchicine, Cucurbitacin B, Cycloheximide, Denatonium benzoate, Diphenidol, Ethylpyrazine, Flavone, N-(3-Oxooctanoyl)-L-homoserine lactone, 6-Propyl-2-thiouracil, N-Phenylthiourea, Sinigrin, Yohimbine |
| dTas2r39 | Acetominophen, Aloin, (-)-α-Thujone, (-)-Camphor, Chloramphenicol, Chloroquine, Colchicine, Denatonium benzoate, Diphenidol, Ethylpyrazine, Flavone, Resveratrol, Sucralose, Thiamine |
| dTas2r40 | Cucurbitacin B, Diphenidol, Ethylpyrazine, Flavone, N-(3-Oxooctanoyl)-L-homoserine lactone, Ofloxacin |
| dTas2r41 | Aloin, (-)-α-Thujone, Aristolochic acid I, (-)-Camphor, Chloramphenicol, Cucurbitacin B, Diphenidol, Flavone, N-(3-Oxooctanoyl)-L-homoserine lactone, Ofloxacin, Oxyphenonium bromide, Sucralose |
| dTas2r42 | Aristolochic acid I, Chloramphenicol, Cucurbitacin B, Diphenidol |
| dTas2r43 | Aloin, (-)-α-Thujone, Aristolochic acid I, Denatonium benzoate, Diphenidol, 6-Propyl-2-thiouracil |
| dTas2r62 | (-)-Camphor, Chloramphenicol, Chlorhexidine, Cucurbitacin B, Cycloheximide, Denatonium benzoate, Diphenidol, N-(3-Oxooctanoyl)-L-homoserine lactone, L-Menthol, Ofloxacin, Thiamine |
| dTas2r67 | Diphenidol, L-Menthol, Thiamine |
