# Supplementary material for: Bitter taste sensitivity in domestic dogs (Canis familiaris) and its relevance to bitter deterrents of ingestion
Source: PLoS One. 2022 Nov 30;17(11):e0277607. doi: 10.1371/journal.pone.0277607 (PMC9710775; doi:10.1371/journal.pone.0277607)
Supplement: Attachment — Submitted filename: Response to reviewers.docx [file pone.0277607.s015.docx]

Bitter taste sensitivity in domestic dogs (*Canis familiaris*) and its relevance to bitter deterrents of ingestion.

Matthew Gibbs^1,5^, Marcel Winnig^2^, Irene Riva^3^, Nicola Dunlop^1^, Daniel Waller^1^, Boris Klebansky^4^, Darren W. Logan^1^, Stephen J. Briddon^5^, Nicholas D. Holliday^5^, and Scott J. McGrane^1^.

1: Waltham Petcare Science Institute, Waltham on the Wolds, Melton Mowbray, Leicestershire, UK.

2: AXXAM GmbH, Constance, Germany.

3: AXXAM SpA, IMAX Discovery Unit, Bresso, Milan, Italy

4: BioPredict, Inc., Demarest, NJ, USA.

5: School of Life Sciences, The Medical School, Queen’s Medical Centre, University of Nottingham, Nottingham, UK.

Corresponding author: [matthew.gibbs@effem.com](mailto:matthew.gibbs@effem.com) (MG)

**Response to reviewers:**

The authors would like to thank the academic editor and reviewers for their time spent reviewing our paper and for the helpful feedback provided. Please see below a point-by-point summary of the changes made to the manuscript. We hope these adequately address the reviewer’s feedback, and we available in case of any further questions.

Journal Requirements:

When submitting your revision, we need you to address these additional requirements.

1. Please ensure that your manuscript meets PLOS ONE's style requirements, including those for file naming. The PLOS ONE style templates can be found at 
<https://journals.plos.org/plosone/s/file?id=wjVg/PLOSOne_formatting_sample_main_body.pdf> and 
<https://journals.plos.org/plosone/s/file?id=ba62/PLOSOne_formatting_sample_title_authors_affiliations.pdf>

This has been addressed in the revised manuscript and supporting information.

2. Please provide information in your manuscript text regarding the source of the animal subjects used in this study.

Please also state in your manuscript text the status of the animal subjects after the research (i.e. rehomed, adopted, housed for further research, euthanized). If any animals were sacrificed, please include information regarding the method of euthanasia and describe any efforts that were undertaken to reduce animal suffering.

These points have been addressed in the revised manuscript.

3. Thank you for stating the following financial disclosure: 
 "All work in the manuscript was funded by Mars Petcare UK. We declare that M.G., N.D., D.W., D.W.L., and S.J.M. are all employees of Mars Petcare UK.
M.G. contributed to the conception and design of the work, the acquisition, analysis, and interpretation of the data, and drafted and substantially revised the work. N.D. contributed to the acquisition, analysis, and interpretation of the data for the in vivo work and revised the draft of the work. D.W. contributed to the analysis and interpretation of the data for the in vivo work and revised the draft of the work. D.W.L. contributed to the conception, experimental design and substantially revised the work. S.J.M. contributed to the conception, experimental design, interpretation of the data for the in vitro and in vivo work, and substantially revised the work."
 
Please state what role the funders took in the study.  If the funders had no role, please state: "The funders had no role in study design, data collection and analysis, decision to publish, or preparation of the manuscript." 
If this statement is not correct you must amend it as needed. 
Please include this amended Role of Funder statement in your cover letter; we will change the online submission form on your behalf.

This has been addressed in the amended cover letter.

4. Thank you for stating the following in the Competing Interests section: 
    "I have read the journal's policy and the authors of this manuscript have the following competing interests: M.G., D.W., D.W.L. N.D., and S.J.M. were all employees of Mars Petcare UK when the research was conducted. I.R. and M.W. are employees of Axxam S.p.A., Italy and Axxam GmbH, Germany.  The study was funded by Mars Petcare UK. M.G. and S.J.M. are named inventors on a patent application on screening methods using canine Tas2r receptors for pet food products and compositions."

Please confirm that this does not alter your adherence to all PLOS ONE policies on sharing data and materials, by including the following statement: "This does not alter our adherence to  PLOS ONE policies on sharing data and materials.” (as detailed online in our guide for authors <http://journals.plos.org/plosone/s/competing-interests>).  If there are restrictions on sharing of data and/or materials, please state these. Please note that we cannot proceed with consideration of your article until this information has been declared.

Please include your updated Competing Interests statement in your cover letter; we will change the online submission form on your behalf.

This has been addressed in the amended cover letter.

5. In your Data Availability statement, you have not specified where the minimal data set underlying the results described in your manuscript can be found. PLOS defines a study's minimal data set as the underlying data used to reach the conclusions drawn in the manuscript and any additional data required to replicate the reported study findings in their entirety. All PLOS journals require that the minimal data set be made fully available. For more information about our data policy, please see <http://journals.plos.org/plosone/s/data-availability>.

"Upon re-submitting your revised manuscript, please upload your study’s minimal underlying data set as either Supporting Information files or to a stable, public repository and include the relevant URLs, DOIs, or accession numbers within your revised cover letter. For a list of acceptable repositories, please see <http://journals.plos.org/plosone/s/data-availability#loc-recommended-repositories>. Any potentially identifying patient information must be fully anonymized.

Important: If there are ethical or legal restrictions to sharing your data publicly, please explain these restrictions in detail. Please see our guidelines for more information on what we consider unacceptable restrictions to publicly sharing data: <http://journals.plos.org/plosone/s/data-availability#loc-unacceptable-data-access-restrictions>. Note that it is not acceptable for the authors to be the sole named individuals responsible for ensuring data access.

We will update your Data Availability statement to reflect the information you provide in your cover letter.

The minimal data set has been supplied as supporting information.

6. Please include your full ethics statement in the ‘Methods’ section of your manuscript file. In your statement, please include the full name of the IRB or ethics committee who approved or waived your study, as well as whether or not you obtained informed written or verbal consent. If consent was waived for your study, please include this information in your statement as well.

This has been included in the manuscript.

7. Please include your tables as part of your main manuscript and remove the individual files. Please note that supplementary tables (should remain/ be uploaded) as separate "supporting information" files (as separate excel or pdf)

This has been addressed in the manuscript.

8. Please review your reference list to ensure that it is complete and correct. If you have cited papers that have been retracted, please include the rationale for doing so in the manuscript text, or remove these references and replace them with relevant current references. Any changes to the reference list should be mentioned in the rebuttal letter that accompanies your revised manuscript. If you need to cite a retracted article, indicate the article’s retracted status in the References list and also include a citation and full reference for the retraction notice.

A number of new references have been added to the manuscript in response to the comments from Reviewer#2.

Reviewer #1: I think the paper is on an interesting issue and the approach is very well structured. Beside being useful about the appropriate use of bitter deterrents to avoid accidental poisonings of pets, could also be useful to understand the evolution of this taste modality in different mammals. Just a small comment: in the introduction Line 41, I suggest to eliminate PRIMARLY. In fact the discovery of extraoral T2Rs and of their functions (metabolic and in innate immunity), open the panorama about the role of bitter taste receptors, as well as of what drove their evolution.
For the rest, the paper is clear and smooth.

We thank Reviewer#1 for their feedback on the manuscript. The word PRIMARILY has been amended to PARTLY on Line 41.

Reviewer #2: The authors used in-vitro and in-vivo tests to deorphanize several canine bitter taste receptors, and then focused on denatonium benzoate and comparison to human sensitivity. This is an important contribution.

Below are my main comments and concerns:

Abstract
“but not in every exposure” – not clear

This text was intended to highlight the fact that in a small minority of cases when dogs were given a choice to drink water or a denatonium solution they would drink more of the denatonium solution. We now feel this is unnecessary in the Abstract and have removed it.


The authors discuss dog-human orthologs. There should be some more in-depth discussion about assignment of dog to human orthologs, and comparison to the mice-human case <https://www.sciencedirect.com/science/article/pii/S0021925820412761?via%3Dihub>

We have included further detail on the assignment of dog-human orthologs including a comparison of chromosomal locations on page 10. Not all dog Tas2rs are annotated in the dog reference genome. Those that are, have been assigned names equivalent to their closest matching human gene. We have followed this convention.

The authors mention the dog genomes database that holds genomes from many dogs from different breeds, and use three different breeds in the behavioral assays. They should explicitly explore the breed or individual variations (if any) in the bitter taste receptors genes.

We completely agree and we have started some investigations on this subject. We hope to publish further on this in the future. We have added some details on the levels of variation we found in dog Tas2rs on page 11. We have started to assess the functional impact of some of these variants in our lab, but work is ongoing.

The age of the dogs varies dramatically (1-9). Distribution of ages should be shown and age should be taken into account in the results.

We have added more detail on the age range of the dogs and done further statistical analysis to assess the impact of age on the data. Details have been added in several places on pages 8, 9, 25 and 31. Age did not show any significant (*p* > 0.05) relationship with intake. This may be because we did not work with extremely young or old dogs, where differences in taste perception may be more apparent.

“ relationship between bitterness and toxicity has been questioned”. The authors cite a seminal paper from 1994(11), but are also encouraged to review an update from the same author <https://link.springer.com/chapter/10.1007/164_2021_451> and a quantification paper <https://pubmed.ncbi.nlm.nih.gov/29130618/>

We agree that these references are important and have added them to the manuscript. We have also adjusted some of the text on page 3.

“All the chimeric receptors retained sensitivity to cucurbitacin B, indicating they were functional.” – but the activation levels in chimeras dropped dramatically, and in some of them there was also a large EC50 shift. These results should be explained and discussed in more detail.

We agree and have added further discussion of these points on pages 15 and 22. In particular, the large increase in the EC50 for the chimeric receptor consisting of hTAS2R10 with dog ECL2 indicates that ECL2 plays a role in cucurbitacin B binding also.

“Insensitivity of dog Tas2r10 to denatonium benzoate is replicated with Gα16/gust44
expressing cells” – this should be also repeated for human Tas2R10 for proper comparison between the two types of in-vitro results. Or at least compared to results for human Tas2R10 activation by denatonium benzoate from the literature.

We do not have data for human TAS2R10 and the Gα16/gust44 cell line currently. We have therefore referenced the published data for hTAS2R10 in more detail as suggested.

Discussion
“Other published studies using similar heterologous cell-based assay systems have reported similar thresholds when comparing in vitro and in vivo data for human receptors” –this may also be relevant to this discussion <https://pubmed.ncbi.nlm.nih.gov/28513558/>

We thank Reviewer#2 for highlighting this reference and have included it in our discussion.

We would like to explain two further updates to the manuscript to the journal editor and reviewers.

While in the process of reviewing our data after we had submitted the manuscript, we came to the conclusion that we should have included an additional dog-human chimera in our experiments. Previously we had not included a dog-human chimera incorporating variable residues in ECL1 and TM3. However, these regions have previously been implicated in TAS2R10 binding and we therefore conducted an additional experiment to include this chimera. The data were consistent with the other chimeras that did not involve ECL2, no sensitivity to DB was detected while low sensitivity to cucurbitacin B was maintained. The data have been incorporated into Fig 2 and the chimera numbering has been adjusted throughout the manuscript.

We would also like to explain some minor changes to the EC_50_ values stated in Table 1. While we previously had two sets of experiments for this in some cases the concentration ranges used in the experiments differed. We decided to repeat some of these experiments to harmonise the concentration range which has resulted in some minor changes to the EC_50_ values stated in the table. This also resulted in some changes to Fig 4 and S2 Fig to reflect the updated data.
